# A Novel Brief Therapy for Patients Who Attempt Suicide: A 24-months Follow-Up Randomized Controlled Study of the Attempted Suicide Short Intervention Program (ASSIP)

**DOI:** 10.1371/journal.pmed.1001968

**Published:** 2016-03-01

**Authors:** Anja Gysin-Maillart, Simon Schwab, Leila Soravia, Millie Megert, Konrad Michel

**Affiliations:** 1 Outpatient Department, University Hospital of Psychiatry, University of Bern, Bern, Switzerland; 2 Translational Research Center, University Hospital of Psychiatry, University of Bern, Bern, Switzerland; 3 Psychiatric Department, General Hospital, Thun, Switzerland; Massachusetts General Hospital, UNITED STATES

## Abstract

**Background:**

Attempted suicide is the main risk factor for suicide and repeated suicide attempts. However, the evidence for follow-up treatments reducing suicidal behavior in these patients is limited. The objective of the present study was to evaluate the efficacy of the Attempted Suicide Short Intervention Program (ASSIP) in reducing suicidal behavior. ASSIP is a novel brief therapy based on a patient-centered model of suicidal behavior, with an emphasis on early therapeutic alliance.

**Methods and Findings:**

Patients who had recently attempted suicide were randomly allocated to treatment as usual (*n* = 60) or treatment as usual plus ASSIP (*n* = 60). ASSIP participants received three therapy sessions followed by regular contact through personalized letters over 24 months. Participants considered to be at high risk of suicide were included, 63% were diagnosed with an affective disorder, and 50% had a history of prior suicide attempts. Clinical exclusion criteria were habitual self-harm, serious cognitive impairment, and psychotic disorder. Study participants completed a set of psychosocial and clinical questionnaires every 6 months over a 24-month follow-up period.

The study represents a real-world clinical setting at an outpatient clinic of a university hospital of psychiatry. The primary outcome measure was repeat suicide attempts during the 24-month follow-up period. Secondary outcome measures were suicidal ideation, depression, and health-care utilization. Furthermore, effects of prior suicide attempts, depression at baseline, diagnosis, and therapeutic alliance on outcome were investigated.

During the 24-month follow-up period, five repeat suicide attempts were recorded in the ASSIP group and 41 attempts in the control group. The rates of participants reattempting suicide at least once were 8.3% (*n* = 5) and 26.7% (*n* = 16). ASSIP was associated with an approximately 80% reduced risk of participants making at least one repeat suicide attempt (Wald χ^2^_1_ = 13.1, 95% CI 12.4–13.7, *p* < 0.001). ASSIP participants spent 72% fewer days in the hospital during follow-up (ASSIP: 29 d; control group: 105 d; *W* = 94.5, *p* = 0.038). Higher scores of patient-rated therapeutic alliance in the ASSIP group were associated with a lower rate of repeat suicide attempts. Prior suicide attempts, depression, and a diagnosis of personality disorder at baseline did not significantly affect outcome. Participants with a diagnosis of borderline personality disorder (*n* = 20) had more previous suicide attempts and a higher number of reattempts.

Key study limitations were missing data and dropout rates. Although both were generally low, they increased during follow-up. At 24 months, the group difference in dropout rate was significant: ASSIP, 7% (*n* = 4); control, 22% (*n* = 13). A further limitation is that we do not have detailed information of the co-active follow-up treatment apart from participant self-reports every 6 months on the setting and the duration of the co-active treatment.

**Conclusions:**

ASSIP, a manual-based brief therapy for patients who have recently attempted suicide, administered in addition to the usual clinical treatment, was efficacious in reducing suicidal behavior in a real-world clinical setting. ASSIP fulfills the need for an easy-to-administer low-cost intervention. Large pragmatic trials will be needed to conclusively establish the efficacy of ASSIP and replicate our findings in other clinical settings.

**Trial registration:**

ClinicalTrials.gov NCT02505373

## Abstract 2

In a randomized controlled trial, Konrad Michel and colleagues test the efficacy of a manual-based therapy intended to prevent repeat suicide attempts.

## Editors' Summary

**Background:**

Suicide is a serious public health problem. Over 800,000 people worldwide die by suicide every year. In the US, one suicide death occurs approximately every 12 minutes. While the causes of suicide are complex, the goals of suicide prevention are simple—reduce factors that increase risk, and increase factors that promote resilience or coping. Factors that increase suicide risk include family history of suicide, family history of child abuse, previous suicide attempts, history of mental disorders (particularly depression), history of alcohol and substance abuse, and access to lethal means. Factors that are protective against suicide include effective clinical care for mental, physical, and substance abuse disorders; connectedness to family and community; and problem solving and conflict resolution skills. A previous suicide attempt is the main risk factor for repeat attempts and for completed suicide. Fifteen to 25 percent of people who attempt suicide make another attempt, and five to ten percent eventually die by suicide.

**Why Was This Study Done?:**

A number of suicide prevention treatments have been developed. Most of them involve therapy sessions and personal follow-up. While some of them have been shown to work in clinical trials—often with participants who have made a previous suicide attempt—few interventions have proven to be effective consistently in different settings. For this study, the researchers developed a treatment called Attempted Suicide Short Intervention Program (ASSIP) composed of three therapy sessions shortly after the suicide attempt and follow-up over two years with personalized mailed letters. They wanted the therapy part to be short, in order to provide a treatment that would allow a psychiatric service to cope with the large number of patients seen in the emergency department after a suicide attempt. The therapeutic elements of the treatment emphasized building an early therapeutic alliance, which would then serve as a basis (“anchoring”) for long-term outreach contact through regular letters. The therapy sessions and letters follow a detailed script, which the researchers developed into a manual that includes a step-by-step description of the highly structured treatment, checklists, handouts, and standardized letters for use by health professionals in various clinical settings. This study was done to test whether ASSIP can reduce suicidal behavior in addition to routine treatment.

**What Did the Researchers Do and Find?:**

The researchers carried out a randomized clinical trial testing ASSIP in people who had attempted suicide (the majority by intentional overdosing) and been admitted to the emergency department of the Bern University General Hospital in Switzerland. Participants were randomly assigned to two groups. The treatment group received ASSIP in addition to treatment as usual (inpatient, day patient, and outpatient care as deemed appropriate by the hospital clinicians); the control group received a single structured assessment interview plus treatment as usual. The study objective was to evaluate—with follow-up questionnaires and health-care data—whether ASSIP can reduce the rate of repeated suicide attempt in the 24 months after a suicide attempt. The researchers also compared suicidal ideation (i.e., whether and how often participants had suicidal thoughts), levels of depression, and how often people were hospitalized between the two groups.

A total of 120 patients who had recently attempted suicide were randomly allocated to treatment as usual or treatment as usual plus ASSIP. The 60 ASSIP participants received three therapy sessions followed by regular contact over 24 months. During the first therapy session, the patient was prompted to tell the story of how he or she had reached the point of attempting suicide. Narrative interviewing is a key element of ASSIP’s patient-centered collaborative approach. The first session was videotaped, and parts were watched and discussed by patient and therapist during the second session, to recreate the experience of psychological pain and analyze how stress developed into suicidal action. During the final session, therapist and patient developed a list of long-term goals, warning signs, and safety strategies. These were printed and given to the patient in a credit-card-sized folded leaflet along with a list of telephone help numbers. Patients were told to carry both items at all times and to use them in the event of an emotional crisis. Over the subsequent two years, patients received six letters from their therapist reminding them of the risk of future suicidal crises and the importance of the collaboratively developed safety strategies.

During the 24 months of follow-up, one death by suicide occurred in each group, five repeat suicide attempts were recorded in the ASSIP group, and 41 repeat suicide attempts were recorded in the control group. ASSIP was associated with an approximately 80% reduced risk of repeat suicide attempt. In addition, ASSIP participants spent 72% fewer days in the hospital during follow-up. There was no difference in patient-reported suicidal ideation or in levels of depression.

**What Do these Findings Mean?:**

The results show that ASSIP, administered in addition to the usual clinical treatment, was able to reduce suicidal behavior over 24 months in patients who had recently attempted suicide. The addition of ASSIP to usual treatment directly or its effect on repeat attempts might also reduce health care costs. The absence of effects on suicidal thoughts and depression is consistent with ASSIP’s objective to help people cope with crises as opposed to eliminating them. The study’s findings in a real-world clinical setting (a university hospital in the Swiss capital) are promising. They justify further testing in large clinical trials and diverse settings to answer conclusively whether and where ASSIP can reduce repeat suicide attempts, prevent deaths from suicide, and reduce health-care costs.

**Additional Information:**

This list of resources contains links that can be accessed when viewing the PDF on a device or via the online version of the article at http://dx.doi.org/10.1371/journal.pmed.1001968.

- National Action Alliance for Suicide Prevention has information on research prioritization for suicide prevention
- There is also a supplemental issue of the *American Journal of Preventive Medicine* focused on research about suicide prevention
- More information about suicide is available from ZEROSuicide http://zerosuicide.sprc.org/ and the Suicide Prevention Resource Center http://www.sprc.org/
- The US Centers for Disease Control and Prevention has information on suicide
- The UK Mental Health Foundation also has information on suicide
- The page “About Suicide” from the American Foundation for Suicide Prevention has information on warning signs, risk factors, and statistics
- The US National Suicide Prevention Lifeline offers help and information
- The Bern University Hospital of Psychiatry has a page describing ASSIP for patients (in German)
- The Finnish Association for Mental Health has a page describing ASSIP (in English)

## Introduction

The high toll of deaths by suicide is a major global public health problem [1]. In spite of a broad range of prevention efforts, suicide rates have not declined [2]. Rates in the US have increased in recent years [3], and the number of suicide attempts in adults has been estimated to be about 1.3 million per year [4]. Individuals with a history of attempted suicide have rightly been identified as a main target group for clinical suicide prevention [5]. A recent meta-analysis reported a 1-year rate of hospital admissions due to repeat attempts of 17%, with a rate of self-reported reattempts of 21.9% [6]. The risk of completed suicide for individuals who have previously attempted suicide is from 40 to over a 100 times higher than that of the general population [7,8]. The risk increases with each subsequent suicide attempt, and remains high for more than 30 years [9]. However, there is a lack of interventions demonstrating a long-term reduction of suicidal behavior [10,11]. Exceptions are the treatment studies by Fleischmann et al. [12], Brown et al. [11], and, most recently, Rudd et al. [13] reporting significant risk reductions over 18, and 24 months. The Fleischmann et al. study was a multicenter study in which 1,867 individuals who had attempted suicide were offered a brief psychoeducative session combined with long-term contact with phone calls or visits over a period of 18 months. The intervention group had significantly fewer deaths from suicide than the group with usual treatment. In the Brown et al. study, which included 120 participants who had attempted suicide, the intervention group received at least ten (up to 24) sessions of cognitive therapy with an emphasis on developing safety plans for future suicidal crises. Case managers ensured treatment adherence of “no show” patients and compliance to ongoing psychiatric treatment. The cognitive therapy group had a significantly lower reattempt rate and a 50% reduced risk of reattempting suicide during 18 months of follow-up. In their study with 152 active-duty soldiers, Rudd and co-workers found that a brief cognitive-behavioral therapy with 12 to 16 or more sessions plus usual mental health care (including case managers) was associated with a 60% lower risk of attempting suicide during 24 months of follow-up. Other studies have found that minimal outreach interventions after attempted suicide, such as contacting patients with regular postcards or standardized letters, are associated with a reduction of suicides [14–16], although findings have not been consistent [17,18]. Considering the limited resources available for the provision of adequate treatment for patients who attempt suicide, there is a clear need for brief, focused, and effective treatments [19].

A major barrier to effective treatment is poor treatment compliance, with 50% or more of patients failing to attend or withdrawing from follow-up treatment within 1 week [20,21]. One reason for this may be that suicidal patients often feel misunderstood by health professionals [22]. According to the traditional medical model, clinicians tend to see suicide as a symptom of mental disorder, while, for people in a suicidal crisis, the central issue is their very personal experience of pain, anguish, hopelessness, and loss of self-esteem [23]. Although adequate treatment of the psychiatric disorder associated with suicidal behavior is important, exploring the very individual, subjective experience of a suicidal crisis requires a patient-centered, collaborative treatment approach [22]. In line with such an approach is the concept of suicide as goal-directed action [24], in which suicide is seen as a solution to a subjectively unbearable mental state. Central to action theory is the notion that people have the narrative competence to explain their actions, i.e., how they reached the point of harming themselves. We previously reported an association between a narrative interviewing style and therapeutic alliance [25].

Therapeutic alliance is a consistent predictor of outcome across psychotherapies [26]. Based on guidelines for clinicians formulated by an international working group [27], we developed a brief therapy program for patients who have recently attempted suicide, the Attempted Suicide Short Intervention Program (ASSIP). A major focus of ASSIP lies in the development of an early therapeutic alliance, combined with psychoeducation, a cognitive case conceptualization, safety planning, and continued long-term outreach contact.

The primary objective of the study was to evaluate the efficacy of ASSIP plus treatment as usual (TAU) compared to TAU alone in reducing the rate of repeated suicide attempts during 24-month of follow-up. Secondary outcome measures were suicidal ideation, depression, and health-care utilization. Furthermore, we investigated the moderating effect of baseline factors such as therapeutic alliance in the therapy sessions, prior suicide attempts, depression at baseline, and diagnosis on outcome.

## Methods

### Design

Patients admitted to the emergency unit of the Bern University General Hospital following attempted suicide were seen on a routine basis by the duty psychiatrist, who asked them for their consent to be contacted by the study team. The signed agreement forms were then forwarded to the study team. Patients who fulfilled the inclusion criteria were randomly assigned to the ASSIP or the control group. Simple randomization was carried out using shuffled unmarked sealed envelopes. Similar to a modified Zelen design [28,29], participants were contacted following randomization and informed that they had been allocated to one of the two groups. Participants in the control group were offered a single assessment interview. This was considered necessary for ethical and safety reasons [30]. Written informed consent was obtained from participants of both groups after detailed description of the study. Suicidality was assessed with the Suicide Status Form (SSF-III) [31] at the initial session. Both groups continued TAU. Study therapists did not have any influence on TAU. A set of questionnaires was filled out by participants after the initial session and at 6, 12, 18, and 24 months.

Local regulations did not require registration of the study before patient recruitment started in 2009. In Switzerland, the Federal Act on Research Involving Human Beings, which requires registration in a public registry, was implemented January 1, 2014. Registration at ClinicalTrials.gov was done after completion of the study. We have not conducted and are not currently conducting any related clinical trials. Future trials will be registered prospectively.

The study procedure was approved by the local ethics committee in accordance with the Declaration of Helsinki [32]. The original project description was evaluated by the research ethics committee of the University of Bern (registration number 144/08, Kantonale Ethikkommission Bern). The trial protocol was submitted on October 9, 2008, resubmitted on April 27, 2009, and accepted on May 22, 2009 (S1 Text).

The study was conducted according to the trial protocol, with minor changes made in the course of the study: (1) the total number of participants in the study protocol (80) was later increased to 120 due to a revised power calculation; (2) time intervals of more than 3 months between suicide attempt and first interview were allowed due to the high number of referrals to the study; (3) habitual self-harm as an exclusion criterion was added later for clarification (see the definition of attempted suicide in “Participants”); (4) in response to peer review, reattempt-free survival, hazard ratios, and group differences were calculated based on multiple imputed datasets.

### Power Analysis and Sample Size

Power calculations were based on the results of previous randomized controlled trials with a comparable design [33,34]. We estimated an expected repetition risk of 30% in the control group, and 15% in the treatment group. We calculated the required sample size for the comparison of survival curves between two groups under the Cox proportional hazards model for clinical trials [35]. For a desired statistical power of 80% to detect a hazard ratio of 0.44 for time to next suicide attempt between treatment groups, with a two-sided alpha level of 0.05, a total sample size of *n* = 116 was required.

### Participants

All participants included in the study had recently attempted suicide. Attempted suicide was defined according to Silverman et al. [36] as a “self-inflicted, potentially injurious behavior with a nonfatal outcome for which there is evidence (either explicit or implicit) of intent to die.” In German-speaking countries, similar to in North America, self-harm is usually defined as non-suicidal self-injury, different from suicidal behavior with intent to die [37]. We therefore deemed it necessary to explicitly exclude habitual self-harm, which is typically associated with a diagnosis of borderline personality disorder (BPD). Further exclusion criteria were serious cognitive impairment, psychotic disorder, insufficient mastery of the German language, and residency outside the hospital catchment area. Information regarding the circumstances of the suicide attempt was collected from the hospital records. For diagnostic information, we used the hospital diagnoses based on the 10th revision of the International Classification of Diseases [38]. Up to four psychiatric diagnoses per participant were recorded.

### ASSIP Treatment Protocol

ASSIP was administered in three 60- to 90-min sessions, usually on a weekly basis. A fourth session was added if necessary. These face-to-face therapy sessions were supplemented by regular, personalized letters to the participants for 24 months. The manual used throughout the study was first published in German [39] in May 2013; the translation in English [40] followed in June 2015. The manual is highly structured, and interventions for each session are described in detail.

#### First session

A narrative interview [41] was conducted in which patients were asked to tell their personal stories about how they had reached the point of attempting suicide. The aim of the narrative interview is to reach—in a biographical context—a patient-centered understanding of the individual mechanisms leading to suicidal behavior and to elicit specific vulnerability factors and trigger events. All interviews were video-recorded, with the patients’ written consent. Suicide risk was assessed using the SSF-III [31].

#### Second session

Patient and therapist, seated side-by-side, watched sequences of the video-recorded first session. The aim of the video playback [42] is to reactivate the patient’s mental state during the suicidal crisis, in a safe environment, and provide a detailed reconstruction of the transition from an experience of psychological pain and stress to the suicidal action. Automatic thoughts, emotions, physiological changes, and contingent behavior were identified. Patients received a psychoeducative handout (“Suicide Is Not a Rational Act”) as a homework task (S2 Text), to be returned with personal comments at the next session. Following the session, the therapist prepared a written draft of the case conceptualization.

#### Third session

The patients’ comments to the handout were discussed. The case conceptualization was revised collaboratively. A list of long-term goals, individual warning signs, and safety strategies was developed in close cooperation with the patient. The written case conceptualization and the personal safety strategies were printed out and given to the patient, with additional copies for other health professionals involved in treatment. Long-term goals, warning signs, and safety strategies were copied to a credit-card-size folded leaflet and given to the patient. In addition, participants received a crisis card with a list of telephone numbers of private and professional helpers who could be contacted in case of a suicidal crisis. Patients were instructed to carry both with them at all times, and to use them in the event of a crisis.

#### Letters

Participants were sent semi-standardized letters over a period of 24 months, every 3 months in the first year and every 6 months in the second year. The letters reminded participants of the long-term risk of future suicidal crises and the importance of the safety strategies. Letters were signed personally by the ASSIP therapist. Usually, one or two personal sentences were added, and participants were invited to give feedback about how things were going.

For further details see ASSIP manual [40].

### Control Group

Participants assigned to the control group underwent a single clinical interview, which included a structured suicide risk assessment using the SSF-III. A written summary of the risk assessment was sent to the health professionals responsible for the participant’s usual clinical care.

### ASSIP Therapists

ASSIP was provided by four therapists: two clinicians experienced in clinical suicide prevention (K. M., psychiatrist, and A. G.-M., psychologist) and two therapists (M. M. and S. Bühler, both psychologists) with an average of 2 years in clinical practice, who were introduced to ASSIP and the study procedures during a 1-week training. Adherence to the highly structured treatment manual, which provides clear tasks for each session, was ensured by regular peer reviews and supervision using the video-recorded interviews.

### Treatment as Usual

Information on the co-active treatment was based on patient’s self-reports in the set of questionnaires completed every 6 months. In both groups, TAU included inpatient, day patient, and individual outpatient care as considered necessary by the clinicians in charge of patient management. Some patients changed from inpatient to outpatient status during ASSIP sessions. Thirty-eight (63%) participants in the ASSIP group and 32 (53%) in the control group were on psychotropic drugs at the time of the initial interview.

### Measures

The set of questionnaires was completed by the study participants at the conclusion of the initial session, and identical questionnaires were sent to both groups every 6 months over a period of 2 years.

#### Personal and sociodemographic characteristics

A 33-item questionnaire was developed to collect sociodemographic and health-related data, including information on suicidal behavior. The questions related to suicidal behavior and self-harm were as follows: (1) “Did you in the past 6 months try to take your own life?”; (2) “How often did you in the past 6 months have thoughts about taking your own life?”; and (3) “How often did you in the past 6 months physically harm yourself (e.g., cutting, burning, hitting)?”. According to the definition of attempted suicide used in this study, physical self-harm during follow-up was not recorded as a suicide attempt. In order to complete outcome data at the end of the follow-up period, we searched hospital records and contacted the responsible general practitioners and therapists to check for suicide attempts and completed suicide.

#### Penn Helping Alliance Questionnaire

The 11-item self-rating Penn Helping Alliance Questionnaire (HAq) [43] was used to evaluate the quality of the patient–therapist relationship (therapeutic alliance). The HAq has demonstrated good validity for psychotherapy outcomes [44]. The German version [45] was used.

#### Beck Depression Inventory

The 21-item Beck Depression Inventory (BDI) was used to measure the severity of depression [46,47].

#### Beck Scale for Suicide Ideation

The Beck Scale for Suicide Ideation (BSS)—a 21-item self-report instrument measuring the intensity of the patients’ attitudes, behaviors, and plans related to suicidal behavior [48,49]—was used.

### Statistical Analysis

Analysis was conducted on the intention-to-treat (ITT) population to ensure unbiased comparisons between the two groups and to address concerns regarding withdrawal and noncompliance. To address the issue of missing data, multiple imputations by chained equations were implemented. Estimates of 20 imputed datasets were combined using Rubin’s rule; the mean and 95% CI of the test statistics and *p*-values are reported. We calculated standard errors and CIs for the Cox hazards coefficients based on the square root of the within-individual variance of the individual imputed datasets and the between-individual variance across the multiple imputed datasets. We found no differences between analyses conducted with the original and the imputed dataset. In the survival analysis, binary events were used (0 = no suicide attempt in the follow-up; 1 = one or more suicide attempts in the follow-up). A *p*-value less than 0.05 was considered statistically significant, and all tests were two-sided. Survival analyses of participants who made a repeat suicide attempt at least once during the follow-up period were conducted using the Kaplan-Meier estimator [50]. Log-rank tests (Mantel-Haenszel test) were performed to calculate survival curve differences between the two groups, and associated Wald χ^2^ tests were conducted to test the null hypothesis of no difference between treatments at any assessment time point. A Cox proportional hazards regression model [51] for discrete data using “exact partial likelihoods” was used to determine hazard ratios for the two groups. Demographic data and secondary outcome measures were analyzed using nonparametric methods (χ^2^ tests, Mann-Whitney *U* test) in the case of count variables (e.g., days of hospitalization, number of suicide attempts) or where data were not normally distributed. BSS and BDI scores were analyzed using ANCOVA. As a sensitivity analysis and to account for potentially autoregressive relationships among scores, we also fitted mixed effects regression models.

All analyses were performed using the SPSS 18.0 software package and R (version 3.2.1; http://www.r-project.org) with the packages “survival” (v. 2.38) and “mice” (v. 2.22).

Data are deposited in the Dryad Digital Repository: http://dx.doi.org/10.5061/dryad.85nf3 [52].

## Results

### Enrollment Statistics

**Fig 1:**
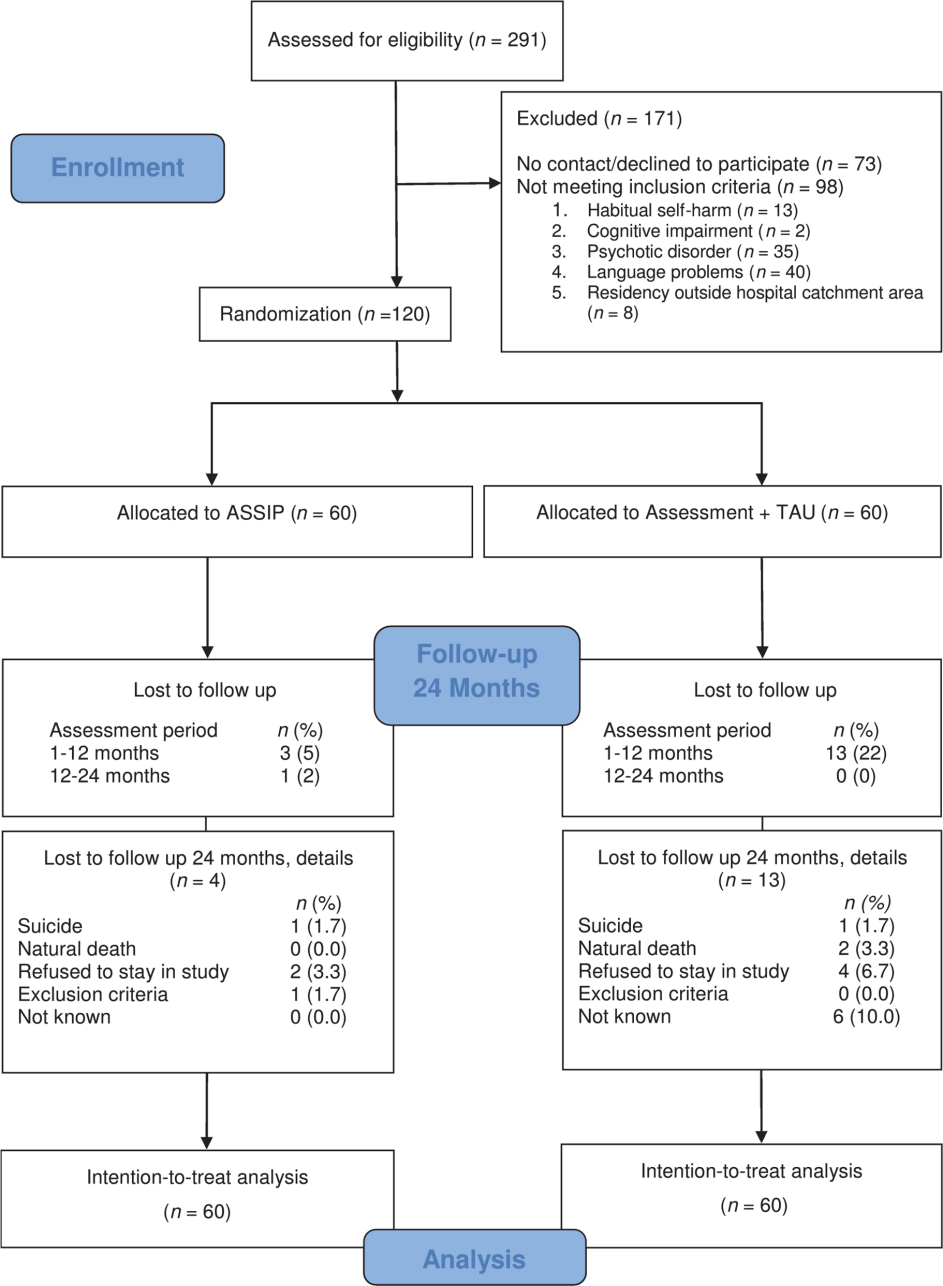
Study flow chart. Flow chart of participants in the randomized controlled trial.

Between June 2009 and December 2012, 291 individuals were referred to the study (see Fig 1); 98 (34%) did not meet the inclusion criteria, and 73 (25%) refused to participate in the study. The most frequent reasons for exclusion were language problems and psychotic disorders. Of those who refused to participate, several denied having had suicidal intention and others refused any kind of treatment or gave circumstantial reasons, such as work commitments and lack of child care, for not being able to attend treatment sessions. Ultimately, 120 patients were randomly assigned to the study groups.

### Participant Characteristics

Fifty-four (45%) male (mean age 40 years, SD = 14) and sixty-six (55%) female (mean age 36 years, SD = 14) participants entered the study (see Table 1). Sixty-three percent were diagnosed with affective disorder, 44% with neurotic and stress-related disorder, and 25% with substance abuse disorder (see Table 1). Prior suicide attempts were reported by 50%; 26% had a history of multiple (two or more) prior suicide attempts. Intentional overdosing was the most frequent method used (68%), followed by cutting (13%), jumping (7%), and hanging, shooting, and drowning (10%). Treatment and control groups did not differ in demographic or clinical variables, with the exception of the number of outpatient sessions prior to the index suicide attempt.

**Table 1:** Baseline demographic and clinical characteristics of study participants.

Characteristic	ASSIP Group (*n* = 60)	Control Group (*n* = 60)	All Participants (*n* = 120)	*p*-Value
**Number of women/men**	36/24	30/30	120	0.36^a^
**Age (years), mean (SD)**	36.5 (14.3)	39.2 (14.6)	37.8 (14.4)	0.32^b^
**Time to first interview (days), median (IQR)**	16.5 (9.0–33.5)	17.5 (9.0–34.5)	17 (9.0–34.3)	0.78^c^
**Diagnosis** ^**d**^ **(ICD-10), *n* (percent)**				0.10^a^
F1*	10 (17%)	20 (33%)	30 (25%)	
F3*	40 (67%)	36 (60%)	76 (63%)	
F4*	25 (42%)	28 (47%)	53 (44%)	
F6*	8 (13%)	12 (20%)	20 (17%)	
Other	6 (10%)	1 (2%)	7 (6%)	
**BDI sum score, mean (SD)**	18.1 (11.4)	18.3 (12.2)	18.2 (11.8)	0.90^b^
**Participants on psychotropic drugs, *n* (percent)**	38 (63%)	32 (53%)	70 (58%)	0.65^a^
**Psychotropic drugs used, *n* (percent)**				
Antidepressants	32 (53%)	24 (40%)	56 (47%)	
Antipsychotics	10 (17%)	11 (18%)	21 (18%)	
Tranquilizers	6 (10%)	9 (15%)	15 (13%)	
Others	7 (12%)	6 (10%)	13 (11%)	
**Married, *n* (percent)**	19 (32%)	15 (25%)	34 (28%)	0.54^a^
**Children, *n* (percent)**	25 (42%)	19 (32%)	44 (37%)	0.34^a^
**Employed, *n* (percent)**	34 (57%)	36 (60%)	70 (58%)	0.85^a^
**Prior suicide attempts, *n* (percent)**				0.071^a^
0	34 (56%)	26 (43%)	60 (50%)	
1	16 (27%)	13 (22%)	29 (24%)	
2 or more (multiple)	10 (17%)	21 (35%)	31 (26%)	
**Lifetime inpatient days, median (IQR)**	21 (0–90)	21 (0–90)	21 (0–90)	0.68^c^
**Outpatient sessions in last 6 months, median (IQR)**	8 (2.25–15.75)	2.5 (0–10.5)	4.5 (0–13.75)	0.010^c^

^a^χ^2^ test.

^b^ *t*-test.

^c^Mann-Whitney *U* test.

^d^Totals exceed 100% because participants had multiple diagnoses. International Classification of Diseases (ICD-10) codes: F1*, substance abuse disorder; F3*, affective disorder; F4*, neurotic and acute stress reaction; F6*, personality disorder.

IQR, interquartile range.

### Attrition Rates and Missing Data

The dropout rates for the ASSIP and control groups were 5% (*n* = 3) and 22% (*n* = 13) at 12 months, and 7% (*n* = 4) and 22% (*n* = 13) at 24 months, respectively. Reasons for withdrawal from the study were, e.g., refusal to cooperate because of change of ASSIP therapist during follow-up, or, in the control group, seeing “too little profit.” In each group, one death by suicide was recorded: one participant in the ASSIP group died by suicide in an acutely psychotic state, and one participant in the control group died by suicide while in hospital care. Three natural deaths occurred in the control group. The number of missed assessments (questionnaires) did not significantly differ between groups (χ^2^_3_ = 7.08, *p* = 0.07), with the largest difference in missed assessments observed at 24 months (ASSIP group: 10%; control group: 37%).

### Primary Outcome Measures

#### Repeated suicide attempts

**Fig 2:**
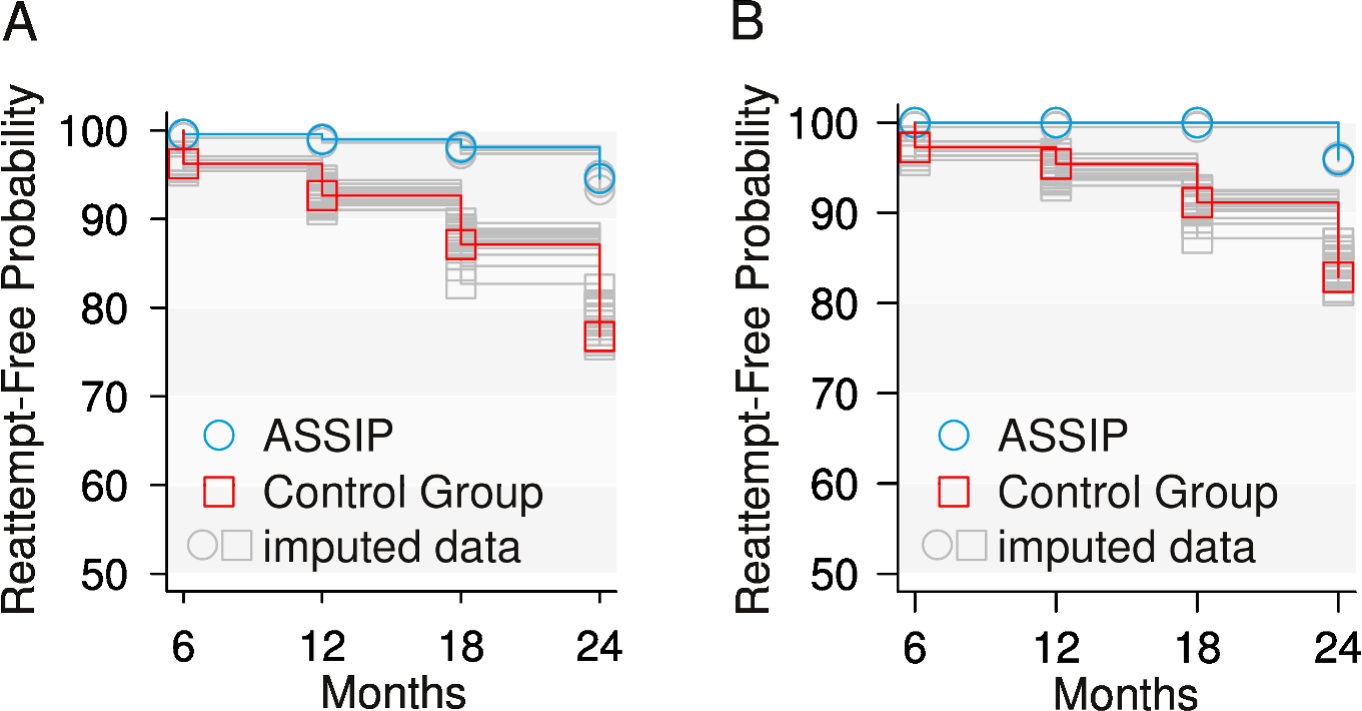
Survival curves. Suicide-attempt-free survival of participants who attempted suicide at least once during the 24-month follow-up period. (A) All participants (*n* = 120). (B) Participants without BPD (*n* = 100).

During the 24-month follow-up period, five reattempts were recorded in the ASSIP group, and 41 reattempts in the control group (see Table 2). The rates of participants reattempting suicide at least once were 8.3% (*n* = 5) and 26.7% (*n* = 16), respectively (ITT analysis). Using the Kaplan-Meier estimator with multiple imputations, the estimated mean suicide-attempt-free survival rate at 12 months was 0.99 (95% CI 0.98–1.00) in the ASSIP group and 0.93 (95% CI 0.89–0.96) in the control group (Fig 2; Table 3). At 24 months, the mean rates were 0.95 (95% CI 0.90–1.00) and 0.79 (95% CI 0.71–0.87), respectively. The group difference in number of participants reattempting suicide was significant (log-rank/Mantel-Haenszel test, mean χ^2^_1_ = 16.5, 95% CI 15.4–17.6; mean *p* < 0.001). The mean hazard ratio was 0.17 (95% CI 0.07–0.46), indicating that the ASSIP group had an 83% reduced risk of attempting suicide during the 24-month follow-up period compared to the control group (Wald χ^2^_1_ = 13.1, 95% CI 12.4–13.7, *p* < 0.001).

**Table 2:** Repeated suicide attempts during 24-month follow up: ITT analysis (*n* = 120).

Follow-Up Period	Group	*N*	Attempts	Persons
**1–6 months**	ASSIP	59	1	1
	CG	52	18	7
**7–12 months**	ASSIP	59	1	1
	CG	50	6	5
**1–12 months**	ASSIP	59	2	2
	CG	43	24	10
**13–18 months**	ASSIP	55	1	1
	CG	42	8	5
**19–24 months**	ASSIP	56	2	2
	CG	42	9	5
**13–24 months**	ASSIP	55	3	3
	CG	40	17	9
**1–24 months**	ASSIP	55	5	5
	CG	43	41	16

ASSIP group: ASSIP therapy plus TAU (*n* = 60); control group (CG): clinical assessment plus TAU (*n* = 60); person sums include individuals with more than one suicide attempt.

**Table 3:** Estimated reattempt-free survival probability during 24-month follow-up.

Assessment Time Point	ASSIP Group		Control Group	
	Reattempt-Free Probability	95% CI	Reattempt-Free Probability	95% CI
6 months	1.00	0.99–1.00	0.96	0.94–0.99
12 months	0.99	0.98–1.00	0.93	0.89–0.96
18 months	0.95	0.96–1.00	0.87	0.82–0.93
24 months	0.95	0.90–1.00	0.79	0.71–0.87

We used a multivariable Cox proportional hazards model to investigate the effect of four baseline covariates on outcome: BDI sum score, previous suicide attempts, diagnosis (substance abuse disorder, affective disorder, neurotic and acute stress reaction, personality disorder), and recent outpatient treatment (number of sessions in the past 6 months). This model revealed a hazard ratio for suicide reattempt for the ASSIP group of 0.15 (95% CI 0.05–0.44, *p* < 0.001) compared to the control group. For the BDI sum score and the diagnosis of personality disorders the hazard ratios were 0.96 (95% CI 0.93–0.99, *p* = 0.016) and 0.39 (95% CI 0.17–0.89, *p* = 0.022), respectively. Prior suicide attempts, a diagnosis of substance abuse, and number of outpatient sessions did not contribute to the model. The overall model showed a significant group difference (Wald χ^2^_8_ = 31.9, 95% CI 30.3–33.4, *p* < 0.001). ASSIP was associated with an 85% reduced risk of participants making at least one repeat suicide attempt. A diagnosis of personality disorder was associated with a 61% higher risk of reattempting suicide, while the BDI sum score increased the risk by 4% per unit.

Furthermore, we tested the effect of a history of multiple suicide attempts on outcome by including “two or more prior attempts” as a covariate. The treatment effect remains, with a hazard ratio of 0.22 (95% CI 0.08–0.61), indicating a 78% reduction of risk due to ASSIP. The overall model is significant (Wald χ^2^_2_ = 23.5, 95% CI 22.4–24.6, *p* < 0.001).

Participants with a diagnosis of BPD had more previous suicide attempts (median = 2, interquartile range [IQR] = 1–2.25) than other participants (median = 0, IQR = 0–1, *W* = 1,450, *p* < 0.001). We therefore separated the 20 participants with BPD from the total sample for additional analysis. For the 100 remaining participants, suicide-attempt-free survival at 24-month was 0.96 (95% CI 0.91–1.00) for the ASSIP group and 0.84 (95% CI 0.76–0.93) for the control group (see Fig 2). The therapy effect was significant (χ^2^_1_ = 12.7, 95% CI 11.8–13.5, *p* < 0.001), with a mean hazard ratio of 0.11 (95% CI 0.03–0.49), indicating that when individuals with BPD were excluded, the ASSIP group had a 89% lower risk of attempting suicide during the 24-month follow-up period than the control group (Wald χ^2^_1_ = 8.7, 95% CI 8.3–9.1, *p* = 0.004).

We additionally tested the sensitivity of the treatment effect. In our model, we predicted the primary outcome, one or more suicide reattempts, by treatment and by previous suicide attempts using a logistic regression. We found a significant effect of treatment (coefficient = 1.66, 95% CI 0.58–2.88, *p* = 0.004), while the effect of previous suicide attempts was not significant (coefficient = 0.20, 95% CI −0.02 to 0.44, *p* = 0.08). This finding is consistent across all imputed datasets (therapy effect, mean *p* = 0.004, 95% CI 0.003–0.006; previous suicide attempts, mean *p* = 0.10, 95% CI 0.07–0.12).

### Secondary Outcome Measures

#### Suicide ideation

Differences in suicide ideation (BSS) during the four follow-up measurements were investigated using a mixed-model ANCOVA on the imputed data. We found a significant reduction in suicide ideation over time (*F*_3,354_ = 8.83, *p* < 0.001; 24-month coefficient: −0.12, 95% CI −0.20 to −0.05), but no group effect (*F*_1,114_ = 0.69, *p* = 0.41). No interaction of group × time was found (*F*_3,354_ = 1.0, *p* = 0.40). Because of differences in diagnoses and number of previous suicide attempts (Table 1), these variables were included as covariates in the ANCOVA, revealing a significant effect of prior suicide attempts (*F*_1,114_ = 17.0, *p* < 0.001; coefficient: 0.05, 95% CI 0.02–0.07) and diagnoses (*F*_1,114_ = 12.8, *p* < 0.001) on BSS scores. Prior suicide attempts correlated positively with BSS scores (Spearman’s rho = 0.27, *p* < 0.001), indicating more suicide ideation in this patient group. These results were supported by using a linear mixed model. We found a significant effect over time (at 18 months: coefficient = −0.07, 95% CI −0.13 to −0.02, *t*_357_ = −2.55, *p* = 0.011; at 24 months: coefficient = −0.14, 95% CI −0.20 to −0.09, *t*_357_ = −5.03, *p* < 0.001), but no group effect (coefficient = −0.02, *t*_114_ = −0.52, *p* = 0.60). Three diagnoses (affective disorder: coefficient = −0.19, 95% CI −0.32 to −0.07, *t*_114_ = −3.01, *p* = 0.003; substance abuse disorder: coefficient = −0.23, 95% CI −0.41 to −0.05, *t*_114_ = −2.48, *p* = 0.014; neurotic and acute stress reaction: coefficient = −0.38, 95% CI −0.52 to −0.23, *t*_114_ = −5.18, *p* < 0.001) and prior suicide attempts (coefficient = 0.05, 95% CI 0.02–0.07, *t*_114_ = 4.12, *p* < 0.001) were significantly associated with BSS scores.

#### Health-care utilization

In the first 12 months, 16 participants (27%) in the ASSIP group and 20 participants (33%) in the control group were admitted to psychiatric inpatient treatment. ASSIP participants had 72% fewer days of inpatient care (median = 29, IQR = 7–68) than control participants (median = 105, IQR = 39–158, *W* = 94.5, *p* = 0.038). At 24 months, 18 (30%) ASSIP participants and 23 (38%) control participants had been admitted to hospital. ASSIP was associated with 63% fewer days of hospitalization (median = 33, IQR = 11–65) than control treatment (median = 90, IQR = 23–180), but the difference at 24 months did not reach significance (*W* = 139.5, *p* = 0.08). The groups did not differ with respect to the total number of outpatient sessions at 12 months (*W* = 1,392.5, *p* = 0.41) and 24 months (*W* = 1,487.5, *p* = 0.57).

#### Depression

Although ASSIP was not expected to have an effect on depression, we analyzed the BDI scores during follow-up using a mixed-model ANCOVA on the imputed data. Similar to the results for the BSS, we found a significant reduction over time (*F*_3,354_ = 18.9, *p* < 0.001), but no group effect (*F*_1,114_ = 1.99, *p* = 0.16). The covariates previous suicide attempts (*F*_1,114_ = 8.5, *p* = 0.004) and diagnoses (*F*_1,114_ = 20.5, *p* < 0.001) had a significant effect on BDI scores; a diagnosis of personality disorder was associated with higher BDI scores. These results were supported by using a linear mixed model. We found a significant effect over time (at 12 months: coefficient = −2.25, 95% CI −3.79 to −0.72, *t*_357_ = −2.89, *p* = 0.004; at 18 months: coefficient = −2.35, 95% CI −3.89 to −0.82, *t*_357_ = −3.02, *p* = 0.003; at 24 months: coefficient = −5.79, 95% CI −7.33 to −4.26, *t*_357_ = −7.44, *p* < 0.001), but no group effect (coefficient = 0.10, *t*_114_ = 0.08, *p* = 0.94). Three main diagnoses (affective disorder: coefficient = −9.76, 95% CI −13.2 to −6.34, *t*_114_ = −5.65, *p* < 0.001; substance abuse disorder: coefficient = −10.5, 95% CI −15.4 to −5.46, *t*_114_ = −4.15, *p* < 0.001; neurotic and acute stress reaction: coefficient = −13.5, 95% CI −17.4 to −9.52, *t*_114_ = −5.18, *p* < 0.001) and prior suicide attempts (coefficient = 0.90, 95% CI 0.29–1.51, *t*_114_ = 2.92, *p* = 0.004) were significantly associated with BDI scores.

### Therapeutic Alliance as Moderating Factor

In the ASSIP group, a linear model demonstrated that therapeutic alliance (HAq score) at the first ASSIP session was inversely related to BSS score at 12 months follow-up (*t*_57_ = −3.02, *p* = 0.004; coefficient: −0.26, 95% CI −0.43 to −0.09, *R* ^2^ = 0.18) and 24 months (*t*_57_ = −3.11, *p* = 0.003; coefficient: −0.21, 95% CI −0.35 to −0.08, *R* ^2^ = 0.30); the relationship between HAq scores at the third ASSIP session and BSS scores was not significant. Furthermore, the ASSIP group showed a significant increase in therapeutic alliance from the first to the third session (HAq at first session, median = 4.91, IQR = 4.3–5.2; HAq at third session, median = 5.32, IQR = 4.9–5.6, *W* = 257.5, *p* < 0.001, paired test).

## Discussion

This randomized controlled trial evaluated the efficacy of ASSIP, a novel brief therapy for individuals who have attempted suicide administered in addition to TAU. The ASSIP group had a 18.4% lower rate of participants making at least one repeat suicide attempt compared to the control group at 24 months, indicating a 83% reduction of the risk of further suicide attempts in the survival analysis. It has been estimated that interventions that reduce suicide attempts by 25% may lead to a 2.6% reduction in the suicide rate, resulting in approximately 1,000 lives saved annually in the US [53]. Emergency department visits and inpatient hospitalizations due to suicidal ideation and suicide attempts have been estimated to result in over 1 million hospital visits per year, leading to costs of US$4.7 billion [1,54]. ASSIP fulfills the demands for a low-cost intervention [55]. Considering the generally limited treatment resources for this patient group, we believe that ASSIP may have a considerable potential for reducing suicidal behavior, increasing treatment provision for patients attempting suicide, and reducing health-care costs. Large pragmatic trials will be needed to conclusively establish the efficacy of ASSIP and replicate our findings in other clinical settings.

A strength of the study is that the intervention was based on a published manual. The treatment protocol is highly structured and, in our experience, easy to understand for therapists as well as for patients. In Finland, ASSIP is currently being used in an increasing number of crisis centers [56]. Thus, we believe that ASSIP can be easily applied in other treatment settings. The study has high external validity due to its real-world setting. Similar to clinical practice, patients seen in the emergency department were informed that referral to ASSIP was part of the routine procedure following attempted suicide. Patients at high risk of suicide were seen for a first interview as soon as they were considered safe for escorted transport to the study premises at the outpatient department. The proportion of participants with a history of one or more prior suicide attempts at baseline was relatively high (50%); other studies report a range of 13% to 24% [57]. This indicates that our sample had a relatively high proportion of individuals with increased suicide risk.

Remarkably, the treatment group spent less time in the hospital during the follow-up period. This may be for two reasons. First, patients in the ASSIP group were often discharged from inpatient care earlier, presumably because the clinicians relied on ASSIP as an effective add-on treatment. Second, the low number of days spent in the hospital by the treatment group probably reflects the reduction of suicide attempts during follow-up.

The treatment effect on suicidal behavior was not reflected in the BSS, measuring primarily suicidal ideation. This finding is consistent with the main objective of ASSIP, which is to implement safety strategies targeting suicidal behavior, and not to reduce suicidality. As part of the therapy interventions, participants were informed that suicidal crises could again be triggered at any time in the future. Furthermore, the results are in line with other studies [11] that found no significant effect of cognitive-behavioral therapy on suicide ideation.

ASSIP is informed by multiple theories and models. But what makes it effective? First, we believe that it is important for patients to know that the goals of the therapy are limited. Patients were informed that they had an increased risk of future suicidal crises and that the therapy would not cure them from suicidality. The declared aim of the therapy was for patients to understand what makes them suicidal, and to develop safety strategies in case of future suicidal crises. The psychoeducative handout is consistent with this aim, promoting the concept of the suicidal mode as an out-of-the-ordinary state of mind that can be triggered at any time by specific adverse experiences [58], and stating that the safety strategies must be applied before the suicidal mode is fully activated. Second, we believe that ASSIP’s patient-centered, collaborative approach to suicidal behavior promotes therapeutic alliance and maximizes treatment engagement, a central element of ASSIP. This is supported by the association of therapeutic alliance measures with outcome and the increase in the scores of therapeutic alliance (HAq) in the ASSIP group between session one and session three. The latter is consistent with results from psychotherapy research, which suggest that ratings of alliance in the third therapy session are a good predictor of adherence to treatment and outcome [59]. Key elements of ASSIP are designed to promote “the active and purposeful collaboration between patient and therapist” [60] and include narrative interviewing [25] and video playback [42], both novel concepts in the treatment of suicidal individuals. Third, ASSIP incorporates established therapeutic concepts, such as safety planning [61] and follow-up contact with regular, personalized letters [14,16]. To our knowledge, so far, no published treatment studies have combined psychological interventions with subsequent ongoing contact through letters. We assume that the regular letters not only provided a feeling of connectedness with the ASSIP therapist, but also may have acted as reminders of patients’ increased suicide risk and may have increased awareness of problems that could trigger future suicidal crises.

There are a number of limitations to be considered. First of all, it has to be taken into account that small trials may result in large effect sizes. Therefore, larger studies will be needed to determine the evidence for the efficacy of ASSIP in reducing suicidal behavior. A problem of treatment studies with long-term follow-up is dropouts and missing data. The cumulative dropout rates increased during follow-up, with higher rates in the control group. This is a well-known phenomenon [54], which was dealt with by ITT analysis, as recommended in the CONSORT guidelines for reporting randomized controlled trial results [55]. The problem of missing data was addressed by using multiple imputations. Multiple imputations reduce bias attributable to missing outcome data and resulted in our study in a more conservative treatment effect compared to the original data.

The groups differed in a few clinical baseline variables, although these differences were not statistically significant, except for the number of outpatient sessions prior to study inclusion. An analysis using a multivariate Cox proportional hazards model with these baseline variables as covariates had no significant influence on the treatment effect: risk reduction for the ASSIP group remained above 80%. The proportion of participants with a history with two or more prior attempts was higher in the control group. However, using “two or more prior attempts” as a covariate did not significantly reduce the treatment effect of ASSIP relative to control treatment.

Information on repeated suicidal behavior was primarily based on participants’ self-reports in the questionnaires. Others [6,33] have reported on the problem of under- and over-reporting in patients’ self-reports of suicidal behavior, and the discrepancy between self-reports and hospital-based data. We dealt with this problem by complementing self-reported data with searching medical records and contacting health professionals involved in TAU.

Data on co-active treatment consisted of self-reports every 6 months on the setting and the duration of TAU. We did not collect information on the nature of the co-active follow-up treatment. Generally, the provision of psychiatric in- and outpatient treatment in the region of Bern can be considered to fulfill high standards of clinical care. The health-care system is a mixture of state and private funding, with mandatory health insurance. Psychiatric outpatient treatment is easily available, with a heavy emphasis on psychotherapy, and with no restrictions on duration. This suggests that the treatment effect cannot be attributed to insufficient co-active treatment.

“Contamination” of ASSIP and control group could not be avoided. Others [62] have commented on the difficulty therapists experience in keeping control and experimental groups “pure.” In our study, initial assessment interviews with the control group were conducted by the therapists who also delivered ASSIP. In practice, the interviews with the control group often included narrative elements. Furthermore, ongoing personal contact with the control group was maintained through the study questionnaires sent to participants with a personally signed letter. In addition, some control participants contacted the therapist for help during the follow-up period. This kind of contamination most likely had the effect of reducing group differences in the primary and secondary outcome measures.

### Comparison with Other Studies and Implications

With three therapy sessions, ASSIP is considerably shorter than any other specific treatments for individuals who have attempted suicide [11,13,33,63,64]. An exception is the treatment in the Fleischmann et al. study [12], in which individuals who had attempted suicide were offered a single psychoeducative session, supplemented by regular outreach contact after discharge. Other effective therapy protocols [11,13] have included elements of active outreach contact (phone calls, home visits). It has been pointed out that outreach elements providing a “lifeline,” ideally based on a therapeutic relationship, may be important for the reduction of suicide risk [65]. In line with this, ASSIP combines face-to-face sessions with regular personalized letters during follow-up. The results support the notion that psychological interventions combined with regular outreach elements may be particularly effective in reducing the risk of further suicidal behavior. Furthermore, the present study suggests that a therapeutic relationship can be established in a brief psychological intervention. The finding that the HAq scores of the first ASSIP session were associated with less suicidal ideation during follow-up suggests that a narrative interview technique may be an important element for establishing an early therapeutic alliance. Other interventions in ASSIP such as cognitive case conceptualization based on a vulnerability–trigger model, psychoeducation, and collaboratively developed crisis response plans are elements used in cognitive-behavioral therapy treatment protocols. A relapse prevention task [38,66] was added to the ASSIP manual as an optional session after completion of the study, and was thus not administered in the present study.

A shortcoming of current therapies is that, to our knowledge, none of the randomized controlled treatment studies so far have been replicated. The probability that a treatment will be implemented and evaluated in other clinical settings will be higher when the therapy protocol is simple and highly structured. We believe that ASSIP fulfills these requirements.

### Conclusions

ASSIP, a manual-based brief therapy for patients who have attempted suicide, administered in a real-world clinical setting, was efficacious in reducing suicidal behavior over 24 months. ASSIP thus fulfills the need for a brief, easy-to-implement, and low-cost intervention. We believe that ASSIP has the potential for dissemination in various treatment settings and for reducing of the costs of health care for patients attempting suicide. Large pragmatic trials will be needed to conclusively establish the efficacy of ASSIP and to replicate our findings in other clinical settings.

## Supporting Information

S1 Text Original trial protocol for ethics committee, May 22, 2009. (PDF)

S2 Text Homework task: “Suicide Is Not a Rational Act.” From: Michel K, Gysin-Maillart A. ASSIP—Attempted Suicide Short Intervention Program: a manual for clinicians. Goettingen: Hogrefe Publishing; 2015. pp. 93–96. http://doi.org/10.1027/00476-000. Reproduced with permission from Hogrefe Publishing (http://www.hogrefe.com)
(PDF)

S3 Text CONSORT checklist of items for reporting trials of nonpharmacologic treatments. (PDF)

## Abbreviations

ASSIP: Attempted Suicide Short Intervention Program
BDI: Beck Depression Inventory
BPD: borderline personality disorder
BSS: Beck Scale for Suicide Ideation
HAq: Penn Helping Alliance Questionnaire
IQR: interquartile range
ITT: intention-to-treat
SD: standard deviation
SSF-III: Suicide Status Form–III
TAU: treatment as usual
