# Supplementary material for: A Novel Brief Therapy for Patients Who Attempt Suicide: A 24-months Follow-Up Randomized Controlled Study of the Attempted Suicide Short Intervention Program (ASSIP)
Source: PLoS Med. 2016 Mar 1;13(3):e1001968. doi: 10.1371/journal.pmed.1001968 (PMC4773217; doi:10.1371/journal.pmed.1001968)
Supplement: S1 Text — (PDF) [file pmed.1001968.s001.pdf]

09.10.2008
rev. 27.04.2009

Study protocol (for ethics committee)
Translated by d. Haessig from the original version in German

Short intervention for patients after attempted suicide

1. Background

After a suicide attempt the risk of a future suicide is increased manifold and for many years to come (Harris & Barraclough, 1997). The risk remains high for many years (Runeson, 2002). The current state of research shows that so far there is no adequate method for treating patients after a suicide attempt that reduces the high suicide risk in the long term. To date various therapies for attempted suicide have been assessed. Only very few studies (Linehan et al., 1991; Brown et al., 2005; Guthrie et al. 2001; Salkovskis et al., 1990) have been able to show that the risk of further suicidal acts could actually be reduced in the 6 (to 24) months following the suicide attempt.
First, this is due to the fact that communication about suicidality often does not take place. Isometsä et al. (1995) for example, found in their study that among 571 cases of completed suicide, the topic of suicide was mentioned only in 22% of cases at the last consultation before the suicide. Among psychiatrists this figure was 39%, among general practitioners 11% and among specialist physicians it was the case in 6% of consultations.
Second, it is a fact that after attempted suicide, patients very often (50% or more) do not take up the offer of follow-up therapy and do not keep their appointments. We can conclude from these two facts that a person at risk of committing suicide needs a special kind of communication and special treatments to be able to talk about his feelings, thoughts and urges to act.

Suicide is an action and not an illness. The traditional medical model of illness is a poor basis from which to understand suicidal patients. In contrast to a traditional medical model, suicide can be understood as a goal-directed action. The perspective of an action theory-based model provides a helpful basis, not only for the understanding, but also for the therapeutic alliance with suicidal patients. Action theory helps us to understand how actions come about and how we can explain and comprehend them. It reveals that human actions can best be understood in the context of hierarchically-organized goal systems. Actions can thus be understood as the expression of goal-oriented actions in which suicide can be seen as a goal, when important topics relating to identity or life are threatened and no alternative action strategy, or indeed, coping strategy is available (Michel & Valach, 1997). In such a situation, in conjunction with an acute life crisis, in addition to life-oriented goals, which accompany us throughout our life, thoughts of suicide may appear as an alternative to life.

Every suicide or attempted suicide has its own story. After an attempted suicide patients report that the suicidal action happened automatically and that they could see no other alternative. A moment of despair, hopelessness and a lack of prospects is typical. A state in which the psychological pain (Israel Orbach, 2003) also known as “mental pain”, is too hard to bear. Suicide therefore appears as a way of putting an end to an unbearable state. Orbach (1994) further found that dissociative states, experienced by suicidal people as their suicidal action unfolds, pave the way for renewed suicide attempts, because an action which is not experienced as causing pain or fear, that even brings relief (an end to mental pain), increases the likelihood that the same plan of action will be activated in similar crises.

Behind every suicidal act is a story. The confiding of a patient’s story can be used for therapeutic purposes. A narrative aims to help patients to tell their own story in their own words. Michel et al. (2004) were able to show that the therapeutic alliance, in interviews with a narrative introduction (“I would like you to tell me in your own words, how it happened...” or “in my opinion, there is a story behind every suicide, can you tell me about it?”), was judged by patients to be significantly better than was the case with structured interviews. This indicates that an interview style based on an action-oriented model, positively influences the therapeutic alliance with suicidal patients.
Suicidal patients usually have good narrative skills, so that the therapist can listen and the suicidal patient can be seen as the expert in his own life story. David Jobes (2006) puts it like this: “I want to see it through your eyes”.
In an acute state of stress, the function of the frontal cortex is impaired, limiting rational planning and actions. This condition is referred to as a suicidal mode (Rudd, 2001).

Therapy for suicidal patients requires specific strategies. It must be assumed that such a suicidal mode – at least after an attempted suicide – cannot be “erased by therapy” because it is stored in the neurons as a solution for an unbearable psychological state. It is available at any time, in particular when a situation, circumstances or difficult feelings threaten one’s self, and no other strategy is present. This leads us to conclude that, in close collaboration with patients, strategies must be developed, which can deter future crises.

The following conclusions can be drawn from the published therapies available:

Conclusions

- Behavioral therapy interventions can be of help (Brown et al., 2005; Linehan et al. 1991, Salkovskis et al. 1991). Patients must learn how to influence these patterns and to stop action patterns from occurring automatically (Linehan et al., 1991).
- The possibility of getting in touch in the event of crises, and outreach contacts by the therapist are helpful (e.g. telephone calls, visiting patients, sending regular standard letters or postcards).
- A good therapeutic alliance, even in a short therapy, is an important element of a effective intervention after attempted suicide.

The background and objective of the study is to bring together the issues mentioned above. Furthermore, by mutual reflection on emotional crises, such as the analysis of suicidal action, important life topics related to the suicidal crisis should be explored.

The purpose of the study is to examine a specific clinical treatment, which aims (a) to explore the background of a suicidal crisis by means of an action-oriented model, (b) to draw up behavior-oriented measures to prevent suicidal acts, and (c) to maintain contact with patients by writing for 2 years in order to have a preventive effect on certain variables of suicidal behavior.
By developing better strategies, together with the availability of a loose but constant therapeutic relationship, we expect to find:

- fewer suicidal acts
- fewer hospital admissions
- better handling of suicidal crises

2. Study objectives

1) Can the behavior of patients who have made one or more suicide attempts be changed in future suicidal crises by a specific short intervention following a suicide attempt?
2) More specifically: Does the intervention reduce the type and frequency of treatments in medical institutions due to suicidal crises (suicidal thoughts, suicide attempts), i.e. are there fewer emergency and regular contacts with health institutions, and is there a decrease in the number of inpatient treatment days?

3. Study design

The short intervention is available in the outpatients clinic of the University Hospital of Psychiatry, University of Bern (UPD). The short intervention's target group are patients after a suicide attempt. The program does not replace any other therapy, to which it should be seen as complementary. The short intervention is available as an outpatient service but is suitable for patients in an outpatient, semi-inpatient or inpatient setting.

Patient recruitment and randomization

The therapy is recommended as a matter of routine by the on-call physician consulted at the UPD, Psychiatric Service, to all patients who have made a suicide attempt or in the context of a (para) suicidal crisis were seen at the psychiatric emergency service of the University Hospital in Bern.

The **procedure** is as follows:

1. After the routine examination of the patient after a suicide attempt by the on-call resident physician responsible, the latter informs the patient that he or she will be called for a follow-up consultation and asks for his written permission for another person to get in touch with him or her for this purpose. The patient is also informed that this is a study offering two different types of therapy.
2. Patients will subsequently receive a telephone call from A. Maillart, Lic. Phil or Prof. Dr. K. Michel (or, if necessary a letter/email) inviting them to a follow-up appointment. In the case of inpatient treatment, prior contact will be made with the physician in charge. As part of standard procedure, when contact is made with patients, they are informed about the therapy by the trial managers (A. Maillart und K. Michel).
3. Ideally, the first appointment should take place within two weeks after the suicide attempt. Other timescales (up to 3 months) are, however, possible. Patients are informed from the start that the treatment is for a short time and that it does not replace regular therapy.

4. Randomization takes place once patients have signed a consent form.

The intervention group then attends 3-4 appointments, which include a narrative interview, video playback and the development of behavioral measures (see point 5). At the end, the patient receives a specially created emergency card, which ensures simple and direct access to the emergency service in the future as well as direct contact with those in charge of the short intervention (Prof. Dr K. Michel, A. Maillart Lic. Phil.). Every 3 months the intervention group receives letters, with another emergency card included. This procedure is continued over a period of two years and provides a loose but continuous therapeutic relationship.

The control group receives a semi-structured clinical interview, which serves as a short analysis and a short report is sent to the physician treating the patient (if they have one). Afterwards, patients receive “treatment as usual“, which is provided by the treating resident physician. The control group is sent the follow-up questionnaire every six months.

4. Study participants

The short intervention is available in the general clinic of the University Psychiatric Service’s (UPD) policlinic in Bern. The short intervention’s target group are patients who are seen after a suicide attempt. Exclusion criteria are: foreign language, imprisonment (secure ward), and acute psychotic disorders. In the case of acute suicidality, the timing of the interview will be discussed with the responsible physician.

Based on published treatment studies we anticipate a large number of patients where the reduction in the number of suicidal acts during follow-up can be proven. Other studies have found a difference in the number of hospitalizations.

Based on a monitoring study which is ongoing in the UPD since 2004, in which all suicide attempts are seen at the psychiatric emergency service of the University General Hospital and the University Hospital of Psychiatry, we anticipate the number of study persons to be N = 80, who will be registered due to a suicide attempt. 40 persons will be randomized to the control group and 40 to the intervention group.

5. Interventions and study protocol

The therapy available in the short intervention project consists of:

1. First consultation: Narrative interview on the background to the suicide. The conversation is video-recorded (with the patient’s written consent).
2. Second consultation: Video playback. Therapist and patient watch the recorded interview together. Regular interruptions are used to reflect and to develop individual

strategies: The background to the suicidal crisis is explored more closely and possible preventive measures are discussed.

3. Third to fourth consultation: Using an action-oriented model, the patterns and processes which precede a suicidal crisis are examined, and possible preventive measures are developed, written down, and handed to the patient.
4. An offer is made to keep in touch in the event of suicidal crises; short intervention crisis card.
5. Agreement to written contact from the study therapist every 3 to 6 months over a period of at least two years (see sample letter in appendix).

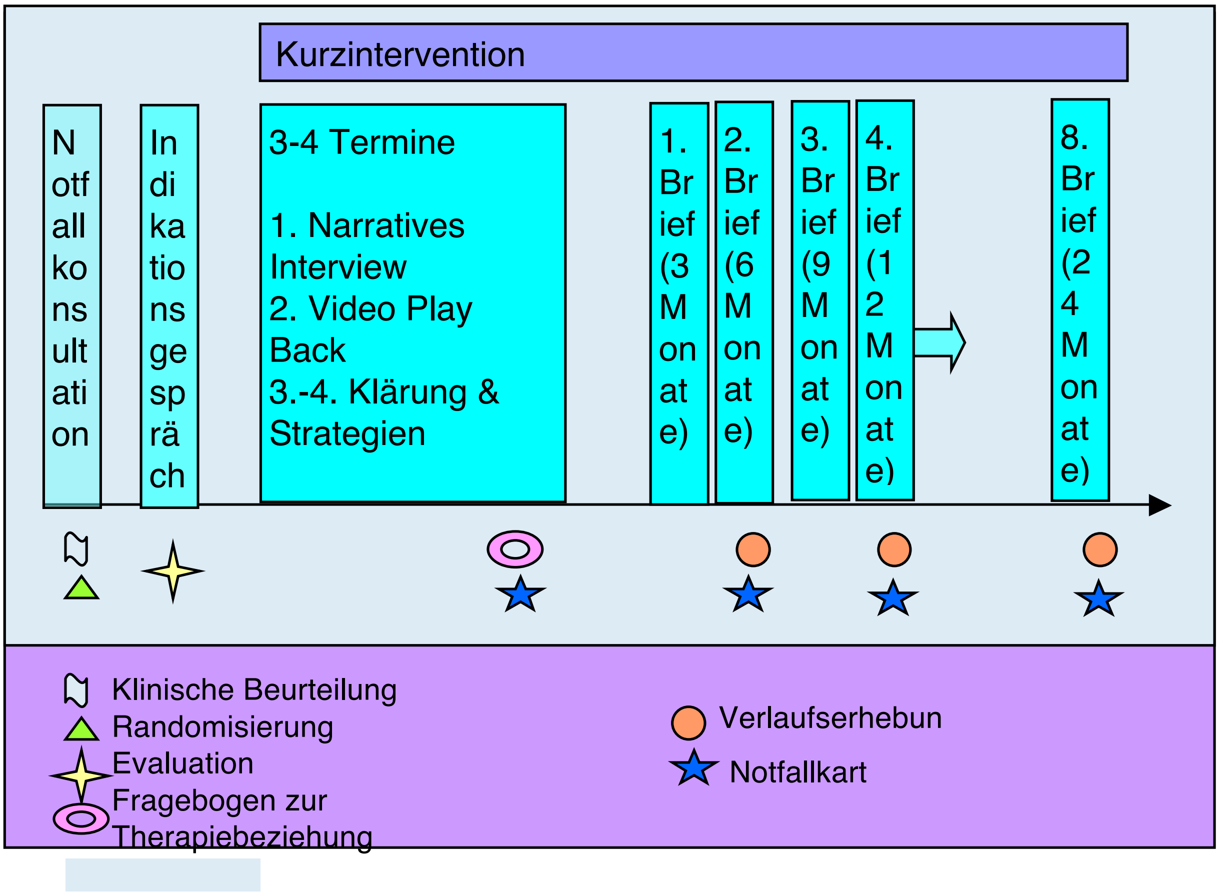

Diagram.1: Flow chart showing short intervention, indication and diagnostic processes.

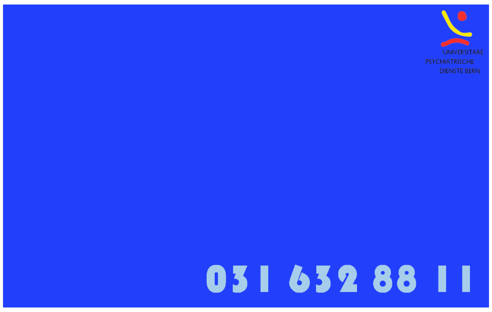

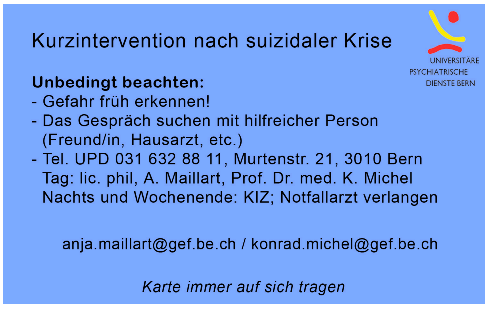

Fig.2: Emergency card for all patients in the study. To ensure easy access to those in charge of the short intervention and to the psychiatric emergency clinic of the University Psychiatric Service, Bern (UPD).

Randomization

At the emergency department or hospital department, all patients are given the same written information with a detachable consent form authorizing contact. They are informed that the therapy available is connected to a follow-up study. The patient receives the information and is asked to sign the consent form authorizing contact. The trial managers receive the signed consent form and contact the patient as part of the follow-up procedure. Randomization takes place when patients are first contacted by Prof. Dr. K. Michel or A. Maillart Lic. Phil, once the patient has given consent to take part in the study.

Patients in the intervention group (IG) receive the short intervention program. Patients in the control group (CG) are given a single appointment, in which (without video) a short assessment (30 minutes) is made, with a summary for the general physician/therapist.

OUTCOME MEASURES

The following variables are measured in the follow-up assessment:

	IG:<br>Short intervention<br>Pre-, post- process evaluation<br>Catamnesis	CG:<br>No short intervention (treatment as usual)<br>Pre-, post-process evaluation,<br>Catamnesis
Number of attempted suicides and suicides		
No. of suicidal crises without action		
No. days inpatient treatment		
No. of outpatient sessions		
No. contacts with therapists		
No. contacts with general practitioner		
No. emergency contacts (incl. emergency dept.)<br>- with general physician<br>- with psychiatrist<br>- emergency dept.		
No. of inpatient days		

Evaluation:

To evaluate the project, patients are asked at the beginning to fill out a questionnaire. As all patients have already been seen by the referring physician, demographic data and diagnoses are already recorded.

The following questionnaires are used:

- Emergency consultation report: Demographic data, diagnoses, history and further procedure
- BDI: The Beck Depression Inventory (BDI) (Beck et al. 1961) is an internationally widespread and generally recognized self-report tool, which is used to measure the severity of depression for over 30 years.
- SSF-II: A self-report tool (short version, adapted as self-report questionnaire). Ranking of self-assessment of the current state with reasons for living, reasons for dying. An authorized German version is available.
- SCL-9: an abridged version of the SCL-90-R, is a tool for measuring the subjective limitation through physical and psychological symptoms.

- Beck Scale for Suicidal Ideation (BSI): is a self-report tool to assess current suicidal thoughts.
- COPE (Carver, 1997) is used to record coping strategies in stressful situations. Follow-up measures: Recording of treatment days, emergency consultations, etc. (diary card).
- Penn Helping Alliance Questionnaire (Alexander & Luborsky, 1990). A self-report tool to measure the strength of the patient-therapist therapeutic alliance. This is used to evaluate the quality of the intervention.

The outcome measurement instruments are deployed as follows:

T1 First consultation short intervention
T2 6 months
T3 12 months
T4 18 months
T5 24 months

6. Risks

There are no risks attached to participation in the short intervention program for patients after attempted suicide. All patients receive treatment as usual.

7. Statistical analyses

By means of repeated measure analyses of variance, the outcome of various parameters (demographic data, therapeutic alliance, suicidal thoughts, mental and physical well-being, etc.) will be analyzed across the measurement times for both groups. Furthermore, predictors for the long-term effect of the short-intervention will be calculated by means of linear regression analyses. The formation of patient groups associated with effective vs. ineffective interventions will be carried out using cluster analysis.

8. References

Alexander, L.B. & Luborsky, L. (1990). *The Penn Helping Alliance Scales.* In L.S. Greenberg and W.M. Pinsoff (Eds.), The psychotherapeutic process: A research handbook (pp. 325-366). New York: Guilford.

Beck, A.T., Ward, C.H., Mendelson, Mock J., Erbaugh, J. (1961). *An inventory for measuring depression.* Archives of General Psychiatry, 4, 561-571.

Beck, A.T., Steer R. A. (1991). Beck Scale for Suicide Ideation. http://www.nimh.nih.gov/suicideresearch/adultsuicide.pdf

Beck, A. T., Kovacs, M., Weissman, A. (1979). Assessment of Suicidal Intention: The Scale for Suicide Ideation. *Journal of Consulting and Clinical Psychology, 47,* 343-352.

Beck, A. T., Steer, R. A., Ranieri, W. F. (1988). Scale for Suicide Ideation: Psychometric Properties of a Self-Report Version. *Journal of Clinical Psychology, 44*, 499-505.

Brown, G.K., Ten Have, T., Henriques G.R., Xie S.X., Hollander, J.E., Beck, A.T. (2005). *Cognitive therapy for the Prevention of Suicide Attempts. A Randomized Controlled Trial.* American Medical Association, 294(5), 563-570.

Carver, C.S. , Scheier, M. F., & Weintraub, J. K. (1989). Assessing coping strategies: a theoretical based approach. *Journal of personality and social psychology, 56 (2),* 267-283.

Carver, C.S. (1997). You want to measure coping but your protocol's too long: consider the brief COPE. *International journal of behavioural medicine, 4,* 92-100.

Franke, G. H. (1995). SCL-90-R. *Die Symptom-Checkliste der Derogatis. German version.* Göttingen: Beltz Test GmbH.

Franke, G. H. (2000). *Brief Symptom Inventory von L.R. Derogatis. German version.* Göttingen: Beltz Test GmbH.

Guthrie, E., Kapur, N., Mackway-Jones, K., Chew-Graham, C., Moorey, J., Mendel, E., et al. (2001). *Randomised controlled trial of brief psychological intervention after deliberate self poisoning*. *British Medical Journal*, 323: 135-138.

Harris, E.C. & Barraclough, B. (1997). *Suicide as an outcome for mental disorders*. British Journal of Psychiatry, 170, 205-228.

Isometsä, E.T., Heikkinen, M.E., Marttunen, M.J., Henriksson, M.M., Aro, H.M., Lönnqvist, J.K. (1995). *The last appointment before suicide: Is suicide intent communicated?* American Journal of Psychiatry,152, 919-922.

Jobes D.A. (2006). *Managing Suicidal Risk: A Collaborative Approach.* Guilford Press. Press.

Linehan M.M., Armstrong H.E., Suarez A., Allmon D., Heard H.L. (1991). *Cognitive behavioural treatment of chronically parasuicidal borderline patients.* Archives of general Psychiatry, 48, 1060-1064.

Michel, K., Valach, L. (1997). *Suicide as goal-directed behaviour*. Archives of Suicide Research, 3, 213-221.

Michel K. (2001). *Suicide is an action - or: we are the agents of our lives*. In: O.T. Grad: Suicide Risk and Protective Factors in the New Millennium (pp 67-71).

Michel, K., Dey, P., Stadler, K., Valach, L. (2004). *Therapist sensitivity towards emotional life career issues and the working alliance with suicide attempters*. Archives of Suicide Research 2004, 8,203-213.

Orbach, I. (1994). *Dissociation, physical pain, and suicide: A hypothesis*. Suicide and Life-Threatening Behaviour, 24, 68-79.

Orbach, I. (2003). *Mental pain and suicide.* The Israel journal of psychiatry and related sciences, 40(3), 191-201.

Orbach I., Mikulincer M., Gilboa-Schechtman E., Sirota P. (2003). Mental Pain and its relationship to suicidality and life meaning. Suicide Life-Threatening Behaviour, 33(3), 231-241.

Rudd, M.D., Joiner, T., Rajab, M.H. (2001). Treating suicidal behaviour. Chapter 2: *A cognitive behavioural model of suicidality*, pp 15-43, New York: Guilford Press.

Runeson, B.S. (2002). *Suicide after parasuicide.* British Medical Journal, 325(7373): 1125-1126.

Salkovskis, P.M., Atha, C., Storer, D. (1990). *Cognitive-behavioural problem solving in the treatment of patients who repeatedly attempt suicide. A controlled trial.* British Journal of Psychiatry. 157, 871-876.

Wagner, B. & Maercker, A. (2007). A 1.5-year follow-up of an Internet-based intervention for complicated grief. *J Trauma Stress* 20(4), 625-9.

9. Appendix

QUESTIONNAIRES

Questionnaires used in the study.

Place / date

Signature: Konrad Michel

Translation
Deborah Haessig
deborah.haessig@bluewin.ch
25/09/2016

Final acceptance letter:

Kantonale Ethikkommission Bern (KEK)
Postfach 56, 3010 Bern

Präsident:
Prof. Dr. pharm. Niklaus Tüller
Email:kek@kek.unibe.ch
www.kek-bern.ch

Generalsekretärin:
Dr. sc. nat. Dorothy Pfiffner
Tel.: 031 632 86 33
Fax: 031 632 86 39
Email:pfiffner@kek.unibe.ch

144/08

Universitäre Psychiatrische Dienste
Prof. Dr. med. Konrad Michel
Oberarzt
Murtenstrasse 21
3010 Bern

Bern, 22.05.09 NT/DP/MM

KEK-Gesuchs-Nr.: 144/08

Kurzintervention für Patienten nach Suizidversuch.
Protocol No. --

Mit Ihrem Schreiben vom 11.05.09 sind folgende Unterlagen eingetroffen:

- Revidiertes Protokoll, 27.04.09, unterschrieben vom Prüfer
- Begleitbrief Patienten dt., nicht datiert.
- Fragebögen dt.: SSF-II, HAQ, SCL-9, Beck-Skala, BDI, Bewältigungsstrategien in stressvollen Situationen, Datenerhebung.

Sehr geehrter Herr Prof. Michel

Besten Dank für die Einreichung der obengenannten Unterlagen.

Die KEK hat das Amendment, die Verwendung zusätzlicher Fragebögen, **präsidialiter geprüft** und **genehmigt**.

Freundliche Grüsse

Ende gut alles gut. Koni

Kantonale Ethikkommission Bern (KEK)

Prof. Dr. pharm. Niklaus Tüller
Präsident

Dr. sc. nat. Dorothy Pfiffner
Generalsekretärin

Gebühr: CHF **0**.-
