# Supplementary material for: A Novel Brief Therapy for Patients Who Attempt Suicide: A 24-months Follow-Up Randomized Controlled Study of the Attempted Suicide Short Intervention Program (ASSIP)
Source: PLoS Med. 2016 Mar 1;13(3):e1001968. doi: 10.1371/journal.pmed.1001968 (PMC4773217; doi:10.1371/journal.pmed.1001968)
Supplement: S2 Text — From: Michel K, Gysin-Maillart A. ASSIP—Attempted Suicide Short Intervention Program: a manual for clinicians. Goettingen: Hogrefe Publishing; 2015. pp. 93–96. http://doi.org/10.1027/00476-000. Reproduced with permission from Hogrefe Publishing (http://www.hogrefe.com) (PDF) [file pmed.1001968.s002.pdf]

Appendix 3. Homework Text: "Suicide Is Not a Rational Act"

Homework – ASSIP

Suicide Is Not a Rational Act

Many of us have moments in our lives in which we may consider suicide as a possible solution to a difficult life situation. This is quite normal. Many people would say that we have this freedom of choice. The state of mind of the acutely suicidal person, however, has little to do with freedom of choice.

Mental Pain

The acute suicidal crisis is usually the result of an experience that fundamentally threatens our sense of self. The associated state of mind is often described as intense psychological or mental pain. This pain may be worse than the most extreme physical pain. Often, it is triggered by a negative experience, such as a threatened or actual breakdown of a relationship, or by an experience of personal failure or loss of important personal goals. The situation becomes dangerous when we start to hate and reject ourselves because of self-blame – that is, when we start to turn against ourselves. When we see no solution to such a painful experience, a state of alarm will ensue, which may be difficult to control. Suicidal people report that they were not their usual self anymore and that they were acting in a trance-like state, that they felt disconnected from their physical body and felt no pain. These critical mental states are called *dissociation,* which means that the normal self-perception is disrupted. In such a condition it is practically impossible to think and act rationally. People lose faith that this experience of alarm and intense pain will ever subside.

Do you personally know such psychological conditions? Please describe your own experience.

The Emotional Brain and the Rational Brain

Recent brain research has begun to shed some light on what happens in the brain in an acute suicidal crisis. Normally, our behavior is the result of the harmonious integration of two major brain regions: on the one hand, the emotional brain, basically located in the limbic system, and on the other, the rational brain, located in the frontal cortex (also referred to as the brain's CEO). The limbic system and, in particular, the amygdala are the brain areas responsible for the detection of threatening situations and for quick behavioral responses. Threatening situations trigger an acute stress response with a release of adrenaline into the blood. This is called a flight–fight reaction, a reaction pattern that can be found throughout evolution. Increased stress hormones (adrenaline and cortisol) drastically reduce the function of the frontal cortical areas, the part of the brain responsible for problem solving, planning, and rational thinking in accordance with our own biography. In a situation of acute emotional stress, these functions are no longer accessible.

This page may be reproduced by the purchaser for personal/professional use.
From: K. Michel & A. Gysin-Maillart: *ASSIP – Attempted Suicide Short Intervention Program* © 2015 Hogrefe Publishing

We would like to know if this makes sense to you. Can you please comment?

Acts of Self-Harm Will Be Stored in the Brain

Once we have experienced a suicidal crisis, particularly after a suicide attempt, such an out-of-the-ordinary emotionally stressful or even traumatic experience will be stored in the brain circuitry as a so-called *suicidal mode*. The suicidal mode is a brain condition learned and stored as a cognitive-emotional-behavioral state of mind, designed to deal with specific, extraordinary situations. This means that it will be reactivated again when triggered by a similar situation. Suicide as a solution will then be a readily available behavioral response to mental pain. It is therefore not surprising that attempted suicide is the main risk factor for later suicide or repeated suicide attempts. Unfortunately, the risk will be elevated for years. This is the reason it is so important to develop and establish effective safety strategies for future crises.

Your comments regarding your own past suicidal crises:

Factors Increasing Suicide Risk

Early traumatic experiences render an individual vulnerable to uncontrollable stress responses in emotionally threatening situations in adult life and therefore increase the risk of suicide. Such traumatic experiences include sexual abuse, maltreatment, violence in the family, but also emotional neglect and separation. They are typically experiences that in childhood and adolescence have already been associated with mental pain, and which left an enduring engram (memory trace). Because the frontal cortex matures up to early adulthood, the adolescent brain in particular is subject to impulsivity and aggression, both risk factors for suicide and attempted suicide.

Is it possible that early negative experiences could be a factor in your case?
Your comments:

This page may be reproduced by the purchaser for personal/professional use.
From: K. Michel & A. Gysin-Maillart: *ASSIP – Attempted Suicide Short Intervention Program* © 2015 Hogrefe Publishing

Depression

Depression is an important risk factor for completed suicide. Depressed individuals tend to be ashamed and blame themselves for their condition. They lose hope that they will get better again. In a state of depression, it is difficult to seek help and talk about the loss of one's usual sense of self. Suicidal thoughts are frequent, and often not communicated.

In depression – which often is the result of emotionally stressful experiences – normal brain function is changed, and this pathological condition cannot be overcome by willpower. In particular, neural activity in the frontal cortex is reduced, problem-solving capacities are impaired, and self-perception tends to be negative ("I am a failure, I am a burden to my family, things will never get better"). All this may lead to the thought that suicide is the only solution. Therefore, it is extremely important to recognize depressive symptoms and to openly talk about suicidal thoughts. Mental health professionals know that depression can be successfully treated, and once people feel better, the suicidal impulses will disappear.

☐ I believe that depression is a factor in my case
☐ I may have other problems with mental health
Please describe:

You Should Know What to Do When You Are Suicidal

The way we deal with an emotional crisis differs individually and depends on a variety of factors. Many people can cope with mental pain, maybe because they have learned to trust that the seemingly unbearable condition will not last forever, but also maybe because their brain is better equipped to deal with extreme stress. However, for most people in times of crisis, it is important to turn to a person in whom they trust and to whom they can talk to about their inner turmoil. If a person has a history of attempted suicide, it is extremely important to be aware of early warning signs and to react before the suicidal mode is switched on, when emotional impulses and dissociation take over. After a suicide attempt it is extremely important to have a list of personal safety strategies, as a means to prevent the dangerous loss of reality. Such lists may include self-help strategies such as walking the dog or going to see a neighbor, but above all, they include names and contact information of persons one can turn to. These may be family members or friends, or it may be a crisis line number, emergency numbers of health professionals, such as a family doctor, psychiatrist, psychologist, etc., who will have the knowledge and experience to help a person to deal with an acute suicidal crisis.

This page may be reproduced by the purchaser for personal/professional use.
From: K. Michel & A. Gysin-Maillart: *ASSIP – Attempted Suicide Short Intervention Program* © 2015 Hogrefe Publishing

In my case, I think the following strategies could be helpful:
1.
2.
3.
4.

Professionals and institutions I could turn to:
1.
2.
3.
4.

Please try to answer the questions, and take this handout to the next session.

Thank you!

<<Name, e-mail address, telephone number of ASSIP therapist>>

This page may be reproduced by the purchaser for personal/professional use.
From: K. Michel & A. Gysin-Maillart: *ASSIP – Attempted Suicide Short Intervention Program* © 2015 Hogrefe Publishing
