# Supplementary material for: A Novel Brief Therapy for Patients Who Attempt Suicide: A 24-months Follow-Up Randomized Controlled Study of the Attempted Suicide Short Intervention Program (ASSIP)
Source: PLoS Med. 2016 Mar 1;13(3):e1001968. doi: 10.1371/journal.pmed.1001968 (PMC4773217; doi:10.1371/journal.pmed.1001968)
Supplement: S3 Text — (PDF) [file pmed.1001968.s003.pdf]

Checklist of Items for Reporting Trials of Nonpharmacologic Treatments*

Section	Item	Standard CONSORT Description	Extension for Nonpharmacologic Trials	Section, paragraph
**Title and abstract†**	1	How participants were allocated to interventions (e.g., "random allocation," "randomized," or "randomly assigned")	In the abstract, description of the experimental treatment, comparator, care providers, centers, and blinding status	Title, Abstract
**Introduction**				
Background	2	Scientific background and explanation of rationale		Introduction, 1-4
**Methods**				
Participants†	3	Eligibility criteria for participants and the settings and locations where the data were collected	When applicable, eligibility criteria for centers and those performing the interventions	Methods, Design, participants
Interventions†	4	Precise details of the interventions intended for each group and how and when they were actually administered	Precise details of both the experimental treatment and comparator	Methods, Treatment conditions
	4A		Description of the different components of the interventions and, when applicable, descriptions of the procedure for tailoring the interventions to individual participants	Methods, ASSIP
	4B		Details of how the interventions were standardized	Methods, ASSIP
Objectives	5	Specific objectives and hypotheses		Introduction, 4
Outcomes	6	Clearly defined primary and secondary outcome measures and, when applicable, any methods used to enhance the quality of measurements (e.g., multiple observations, training of assessors)		Introduction, 4
Sample size†	7	How sample size was determined and, when applicable, explanation of any interim analyses and stopping rules	When applicable, details of whether and how the clustering by care providers or centers was addressed	Methods, 3 Power Analysis and Sample Size
Randomization–sequence generation†	8	Method used to generate the random allocation sequence, including details of any restriction (e.g., blocking, stratification)	When applicable, how care providers were allocated to each trial group	Methods, Design

Allocation concealment	9	Method used to implement the random allocation sequence (e.g., numbered containers or central telephone), clarifying whether the sequence was concealed until interventions were assigned		Methods, Design
Implementation	10	Who generated the allocation sequence, who enrolled participants, and who assigned participants to their groups		Methods, Design
Blinding (masking)†	11A	Whether or not participants, those administering the interventions, and those assessing the outcomes were blinded to group assignment	Whether or not those administering co-interventions were blinded to group assignment	n/a
	11B		If blinded, method of blinding and description of the similarity of interventions†	
Statistical methods†	12	Statistical methods used to compare groups for primary outcome(s); methods for additional analyses, such as subgroup analyses and adjusted analyses	When applicable, details of whether and how the clustering by care providers or centers was addressed	Methods, Statistical Analysis
**Results**				
Participant flow†	13	Flow of participants through each stage (a diagram is strongly recommended)---specifically, for each group, report the numbers of participants randomly assigned, receiving intended treatment, completing the study protocol, and analyzed for the primary outcome; describe deviations from study as planned, together with reasons	The number of care providers or centers performing the intervention in each group and the number of patients treated by each care provider or in each center	Results, Figure 1
Implementation of intervention†	New item		Details of the experimental treatment and comparator as they were implemented	Methods, Treatment conditions
Recruitment	14	Dates defining the periods of recruitment and follow-up		Results, Enrollment statistics
Baseline data†	15	Baseline demographic and clinical characteristics of each group	When applicable, a description of care providers (case volume, qualification, expertise, etc.) and centers (volume) in each group	Results, Table 1

Numbers analyzed	16	Number of participants (denominator) in each group included in each analysis and whether analysis was by "intention-to-treat"; state the results in absolute numbers when feasible (e.g., 10/20, not 50%)	å	Fig. 1<br>Table 2
Outcomes and estimation	17	For each primary and secondary outcome, a summary of results for each group and the estimated effect size and its precision (e.g., 95% confidence interval)		Results, Primary Outcome Measures, Secondary Outcome Measures
Ancillary analyses	18	Address multiplicity by reporting any other analyses performed, including subgroup analyses and adjusted analyses, indicating those prespecified and those exploratory		Results, Repeated Suicide Attempts
Adverse events	19	All important adverse events or side effects in each intervention group		Results, Attrition Rates and Missing Data
**Discussion**				
Interpretation†	20	Interpretation of the results, taking into account study hypotheses, sources of potential bias or imprecision, and the dangers associated with multiplicity of analyses and outcomes	In addition, take into account the choice of the comparator, lack of or partial blinding, and unequal expertise of care providers or centers in each group	Discussion, 1,2,3
Generalizability†	21	Generalizability (external validity) of the trial findings	Generalizability (external validity) of the trial findings according to the intervention, comparators, patients, and care providers and centers involved in the trial	Conclusions
Overall evidence	22	General interpretation of the results in the context of current evidence		Discussion, Comparison with other studies and implications

*Additions or modifications to the CONSORT checklist. CONSORT = Consolidated Standards of Reporting Trials.
†This item was modified in the 2007 revised version of the CONSORT checklist.
